# High Biocompatibility, MRI Enhancement, and Dual Chemo- and Thermal-Therapy of Curcumin-Encapsulated Alginate/Fe_3_O_4_ Nanoparticles

**DOI:** 10.3390/pharmaceutics15051523

**Published:** 2023-05-18

**Authors:** Xuan-Hai Do, Tu Dac Nguyen, Thi Thu Huong Le, Thuy Thanh To, Thi Van Khanh Bui, Nam Hong Pham, Khanh Lam, Thi My Nhung Hoang, Phuong Thu Ha

**Affiliations:** 1 Department of Practical and Experimental Surgery, Vietnam Military Medical University, 160 Phung Hung Road, Ha Dong District, Hanoi 10000, Vietnam; 2 Vinmec Center of Applied Sciences, Regenerative Medicine, and Advance Technologies, 458 Minh Khai, Hai Ba Trung District, Hanoi 10000, Vietnam; v.tund5@vinmec.com (T.D.N.); hoangthimynhung@hus.edu.vn (T.M.N.H.); 3 Faculty of Biology, VNU University of Science, Hanoi, 334 Nguyen Trai Road, Thanh Xuan District, Hanoi 10000, Vietnam; tothanhthuy@hus.edu.vn (T.T.T.); buithivankhanh@hus.edu.vn (T.V.K.B.); 4 Institute of Materials Science, Vietnam Academy of Science and Technology, 18 Hoang Quoc Viet Road, Cau Giay District, Hanoi 10000, Vietnam; lehuongmaket@gmail.com (T.T.H.L.); namph.ims@gmail.com (N.H.P.); 5 Department of Chemistry, Faculty of Natural Resources and Environment, Vietnam National University of Agriculture, Trau Quy, Gia Lam District, Hanoi 12400, Vietnam; 6 108 Military Central Hospital, 1 Tran Hung Dao Road, Hai Ba Trung District, Hanoi 10000, Vietnam; lamkhanh.himed@gmail.com

**Keywords:** magnetic nanoparticles, MRI, hyperthermia, cytotoxicity, curcumin, sarcoma

## Abstract

(1) Background: Magnetite (Fe_3_O_4_) nanoparticles have great potential for biomedical applications, including hyperthermia and magnetic resonance imaging. In this study, we aimed to identify the biological activity of nanoconjugates composed of superparamagnetic Fe_3_O_4_ nanoparticles coated with alginate and curcumin (Fe_3_O_4_/Cur@ALG) in cancer cells. (2) Methods: The nanoparticles were evaluated for the biocompatibility and toxicity on mice. The MRI enhancement and hyperthermia capacities of Fe_3_O_4_/Cur@ALG were determined in both in vitro and in vivo sarcoma models. (3) Results: The results show that the magnetite nanoparticles exhibit high biocompatibility and low toxicity in mice at Fe_3_O_4_ concentrations up to 120 mg/kg when administered via intravenous injection. The Fe_3_O_4_/Cur@ALG nanoparticles enhance the magnetic resonance imaging contrast in cell cultures and tumor-bearing Swiss mice. The autofluorescence of curcumin also allowed us to observe the penetration of the nanoparticles into sarcoma 180 cells. In particular, the nanoconjugates synergistically inhibit the growth of sarcoma 180 tumors via magnetic heating and the anticancer effects of curcumin, both in vitro and in vivo. (4) Conclusions: Our study reveals that Fe_3_O_4_/Cur@ALG has a high potential for medicinal applications and should be further developed for cancer diagnosis and treatment.

## 1. Introduction

Nanoparticles (NPs) possess unique chemical and physical properties and play important roles in various research fields, including electronics, energy, and biomedicine. Among the numerous types of NPs, magnetic NPs show great promise because of their unique properties in magnetic fields and lack of a depth-penetration limit in the human body [1,2,3,4]. NPs have many potential biomedical applications, including magnetic drug targeting, hyperthermia, cell separation, and magnetic resonance imaging (MRI) [5,6,7,8,9,10,11]. They have also been shown to be effective in cancer diagnoses and treatment [5,6,11,12,13,14,15] or have antibacterial activity with low mammalian toxicity [16]. 

Among the magnetic iron oxide nanoparticle family, magnetite (Fe_3_O_4_) nanoparticles (MNPs) are some of the most popular NPs because of their high chemical stability and better magnetic strength compared to other popular iron oxide nanoparticles, such as maghemite (γ-Fe_2_O_3_) and hematite (α-Fe_2_O_3_) [16,17]. In addition, MNPs possess superparamagnetic properties at the nanoscale level [16,17,18]. Thus, MNPs are the most common iron oxide nanoparticles employed in biomedical applications [16]. Several methods have been developed for synthesizing MNPs, including coprecipitation, solvothermal methods, [17] wet-chemical reduction [19], micro/nanoemulsion [13], sonochemical or sonolysis methods [20], and green and extraction–pyrolytic methods [21]. Each method has its advantages and disadvantages; however, all of them could synthesize nanoparticles with suitable characteristics for biomedical applications, including magnetic properties, dispersity, size, size distribution, and shape control. In particular, the size and size distribution of MNPs play major roles in the heating efficacy and MRI contrast [5,22].

Biocompatibility and toxicity are two main areas of concern when using MNPs for clinical applications. When MNPs enter the cell, they can affect nuclear activity or cause cell membrane leakage or blockage, leading to decreased cell proliferation, viability, and metabolic activity [23]. The surface functionalization of magnetic NPs can improve their dispersion and biochemical characteristics, including biocompatibility [14,24,25]. Alginate is one of the most common natural polymers with advantageous properties for functionalizing Fe_3_O_4_ NPs [26]. In addition, combinations of MNPs with anticancer drugs, such as paclitaxel, doxorubicin, docetaxel, and curcumin, are considered a promising strategy for the development of MNPs as multifunctional nanosystems for biomedical applications [7,25,27,28,29]. Among these compounds, curcumin has attracted the most attention from scientists due to its anticancer activity and drug-labeling capacity. Several publications have reported the combination of MNPs and curcumin in inducing cancer cell death, cellular uptake, and biodistribution monitoring [7,10,30]. In 2014, Devkota et al. engineered multifunctional magnetic nanoconjugates composed of superparamagnetic Fe_3_O_4_ NPs coated with alginate and curcumin [12]. They show that this nanosystem is promising for applications in hyperthermia and biomolecular detection. However, most of these studies were performed in vitro, and very few have been performed in vivo. In addition, the studies lack a toxicity evaluation of the nanoparticles on the experimental animals. Moreover, there is little correlation between in vitro and in vivo studies because of the inability of in vitro studies to mimic complex environments in vivo. Hence, many in vitro results have not been translated to in vivo models [31,32].

In this study, we aimed to identify the acute and sub-chronic toxicity and biodistribution toxicity of the nanoconjugates composed of superparamagnetic Fe_3_O_4_ NPs coated with alginate and curcumin (Fe_3_O_4_/Cur@ALG) in mice. After that, the bioeffects of the nanoconjugates were evaluated using both in vitro and in vivo models. MRI contrast enhancement and efficient treatment of hyperthermia and curcumin combination of the nanoconjugates were demonstrated.

## 2. Materials and Methods

### 2.1. Materials

Curcumin, alginate, ferric chloride, and ferrous chloride tetrahydrate were purchased from Sigma-Aldrich (St. Louis, MO, USA). Solvents (NH_4_OH [26% ammonia] and ethanol) were purchased from Merck (Darmstadt, Germany). Distilled water was used for nanoparticle preparation experiments.

### 2.2. Synthesis of Fe_3_O_4_@Alginate Iron Oxide Nanoparticles

Ha et al. [18] produced Fe_3_O_4_ magnetic nanoparticles by co-precipitating ferric and ferrous hydroxides under alkaline conditions. All experiments employed distilled water, hereafter referred to as water. The reaction equations are as follows:

2Fe^3+^ + Fe^2+^ + 8OH^−^ → Fe_3_O_4_ + 4H_2_O

Following a typical process, 4 mmol (0.65 g) of FeCl_3_ and 2 mmol (0.3977 g) of FeCl_2_·4H_2_O were dissolved in 50 mL of 2 M HCl and poured into a 250 mL three-necked flask with a Teflon-coated magnetic stir bar. The mixture was agitated aggressively under N_2_ purging. Throughout the procedure, the temperature was maintained at 70 °C. When 80 mL of 2 M NH_4_OH was added to the reaction mixture, a precipitate formed, turning the entire solution black and increasing the pH to 9. After 30 min of churning, the product was rinsed numerous times with water until it reached a pH of 7. The finished product was washed with ethanol and dried at 60 °C for 24 h for characterization, or it was dispersed in water for further surface functionalization, henceforth referred to as Fe_3_O_4_.

Fe_3_O_4_ nanoparticles were distributed in water at a concentration of 3 mg/mL and coated with alginate. Next, 0.1 g of alginate was added to 10 mL of water and agitated for 2 h until thoroughly dissolved. Subsequently, 30 mL of the Fe_3_O_4_ suspension was gently dropped into the alginate solution at room temperature under ultrasonication. Subsequently, the Fe_3_O_4_ nanoparticles and alginate combination were magnetically swirled for 48 h. Finally, the uncoated particles were centrifuged to yield a magnetic fluid containing 5 mg/mL of Fe_3_O_4_ nanoparticles coated with alginate (Fe_3_O_4_@ALG).

According to Ha et al. [33], curcumin (Cur) was integrated into the magnetic system of Fe_3_O_4_@ALG nanoparticles. First, a 200 mL flask with a magnetic stir bar was filled with 20 mL of 5 mg/mL Fe_3_O_4_@ALG magnetic fluid. Cur (0.1 g) was dissolved in 15 mL of ethanol and placed in the Fe_3_O_4_@ALG magnetic fluid in a separate container. The mixture was agitated for 48 h in a closed jar before the ethanol was removed. To eliminate unencapsulated Cur, the resultant product was centrifuged at 5000 rpm for 5 min. The unencapsulated Cur was dried and weighted, to be around 64 mg. Therefore, the amount of encapsulated Cur in the magnetic fluid was 36 mg. The Fe/Cur mass ratio in the sample was calculated to be 2:1. The final product (Fe_3_O_4_/Cur@ALG) was stored at room temperature until further use.

### 2.3. Material Characterization

Structural characterization was performed using Fourier-transform infrared spectroscopy (FTIR, SHIMADZU spectrophotometer) with KBr pellets within the wavenumber region 400–4000 cm^−1^. X-ray diffraction (XRD) patterns were determined with a SIEMENS D5005 diffractometer using Cu-Kα radiation at 0.15406 nm. The diffraction patterns were collected within a 2θ range of 20°–70°. The solid-state particle size of the NPs was observed using transmission electron microscopy (TEM, JEM 1010). The hydrodynamic particle size and zeta potential distributions of the samples were determined using a Zetasizer-Nano ZS device (Malvern, UK) through the dynamic laser scattering method.

### 2.4. Cell Culture and Cell Viability Assay

Sarcoma 180 cancer cells were cultured in RPMI 1640 media (Gibco) containing 10% FBS (Gibco) and 1% penicillin/streptomycin (Invitrogen) and incubated at 5% CO_2_, 37 °C. An MTT cell proliferation assay kit (Promega) was used to evaluate the cytotoxicity of Fe_3_O_4_@ALG and Fe_3_O_4_/Cur@ALG. A total of 5 × 10^6^ cells were exposed to Fe_3_O_4_ concentrations ranging from 0.01 to 1000 µg/mL for 48 h. The samples were measured using laminator spectrometers at a wavelength of 570 nm. The IC50 (the half maximal inhibitory concentration) value was calculated according to the guidelines of the MTT assay kit.

### 2.5. Immunofluorescence Assay

Cells were grown on Labtek chamber slides (Nunc) in RPMI 1640 culture medium and incubated with Fe_3_O_4_/Cur@ALG at concentrations ranging from 0.01 to 1000 µg/mL for 48 h. The medium was removed via centrifugation and the cells were washed with phosphate-buffered saline (PBS). Cells were fixed with 4% paraformaldehyde (Gibco) and 2% sucrose (Sigma, Hà Nội, Vietnam) for 15 min at 37 °C, permeabilized with 0.2% Triton X-100 (Sigma) for 10 min, blocked with 5 mg/mL BSA (Sigma), incubated with mouse monoclonal anti-human α-tubulin antibodies (Invitrogen) for 1 h, and incubated with Alexa 546-conjugated anti-mouse antibodies for 30 min. DNA was visualized using Hoechst 33,342 (Invitrogen). Images were obtained using a ZEISS 510 laser-scanning confocal (LSM) microscope.

### 2.6. Animal Experiments

Swiss mice (5–7 weeks old) were obtained from the Standard Animal Research and Production Center of the National Institute of Hygiene and Epidemiology (Vietnam). The animals were housed in clean polypropylene cages and maintained in an air-conditioned conventional animal house at 25 °C, 55% relative humidity, and a 12 h/12 h light/dark cycle. 

### 2.7. Toxicity Test

Acute and sub-chronic toxicity tests were performed in accordance with the Organization for Economic Co-operation and Development (OECD) guideline 425 [34], and the principles and procedures outlined in the national regulations.

For the acute toxicity test, male Swiss mice (5–7 weeks old) were intravenously injected with Fe_3_O_4_/Cur@ALG NPs at different doses (80, 90, 100, 110, and 120 mg/kg; n = 5 mice for each dose). The mice were observed continuously for 72 h.

To evaluate sub-chronic toxicity, mice were intravenously injected with Fe_3_O_4_/Cur@ALG NPs at two doses (24 and 17 mg/kg; n = 10 mice for each dose) every 48 h for 30 days. The control mice were injected with PBS. The mice were observed for any adverse reactions, such as death, changes in body weight, food consumption, stool, and motor activity. The body weights of the mice were measured every 72 h. On day 31, all mice were sacrificed, and blood samples were obtained for serum chemistry analysis, including alanine aminotransferase (ALT), aspartate aminotransferase (AST), blood urea nitrogen (BUN), and creatinine. Meanwhile, the liver and kidney tissues were collected for histological analyses. The samples were fixed in 4% formalin for 48 h and then mass-casted with paraffin, cut into 7 µm-thick slices, and stained with hematoxylin–eosin (Sigma, USA). The tissue characteristics and morphology were examined by pathologists using optical microscopy.

### 2.8. The Lifetime of Fe_3_O_4_/Cur@ALG NPs in the Blood

The lifetime of Fe_3_O_4_/Cur@ALG NPs in the blood was determined using magnetic inductive heating experiments. A generator (RDO HF1, 5 kW) was used to create an AMF with an amplitude of 80 Oe and a frequency of 178 kHz. Male Swiss mice (n = 5) received 1000 µg of Fe_3_O_4_/Cur@ALG via the tail vein. At 10 min, 2 h, 12 h, 24 h, and 48 h post-injection, blood was collected, placed in a round-bottom-shaped glass holder, and subjected to the AMF. The temperatures of the samples were measured using an optical thermometer (Opsens).

The concentration of Fe in the blood of the mice was also determined by atomic absorption spectrometry (AAS) using a Shimadzu 6300 AAS AA/AE Spectrophotometer. Briefly, blood samples were placed in a Kjeldahl bottle with 10 mL of H_2_SO_4_ and mixed thoroughly. The bottle was heated to 450 °C for 15 min, after which several drops of 70% perchloric acid were added. When the solution turned transparent, it was ready for AAS analysis. The Fe content in the blood samples is calculated as a = cV/m, where c is the concentration of Fe in the sample (kg/L), v is the volume of the sample (L), and m is the weight of the sample (kg).

### 2.9. In Vitro Magnetic Resonance Imaging (MRI)

Fe_3_O_4_/Cur@ALG was homogeneously dispersed in 1% agarose (Invitrogen) prepared in two solutions: (1) PBS (Gibco) and (2) PBS containing 1 × 10^6^ sarcoma 180 cells. MRI of the samples was performed using a Phillips Gyroscan 3.0 T MRI scanner (Philips, Eindhoven, Netherlands). T1- and T2-weighted imaging were performed using the following parameter settings: TR = 3000 ms, TE = 80 ms, and FA = 90°. The brightness signal of the MRI was converted into pixels using ImageJ software.

### 2.10. In Vitro Hyperthermia Assay

A total of 10^6^ sarcoma 180 cells were dispersed in PBS containing Fe_3_O_4_/Cur@ALG with 5 concentrations of Fe_3_O_4_, ranging from 100–2000 µg/mL. A generator (RDO HFI, 5 kW) was used to create an alternating magnetic field (AMF) with an amplitude and frequency of 80 Oe and 178 kHz, respectively. After the solution reached the maximum temperature at each Fe_3_O_4_ concentration, the magnetic field was maintained for 30 and 45 min. The percentage (%) cell viability was determined using the Trypan blue (Sigma) staining technique at 0, 10, 20, 30, and 60 min after applying an AMF. 

### 2.11. In Vivo MRI

Male Swiss mice were subcutaneously injected with 2 × 10^6^ sarcoma 180 cells in the left side of the chest. At day 5 post-implantation, when the tumor size had reached an average volume of 100 mm^3^, the Fe_3_O_4_/Cur@ALG nanoparticle solution was applied to each mouse via subcutaneous injection (at two Fe concentrations of 50 µg and 250 µg per tumor) or via the tail vein (at a Fe concentration of 100 µg/mouse). Control mice were not transplanted with cancer cells, and tumor controls were not injected with NPs. The mice were anesthetized via intraperitoneal injection of thiopental (50 µg/kg) (Rotexmedica, Germany). MRI was conducted on a commercial Phillips Gyroscan 3.0 T MRI system with the following parameters: TR, 3000 ms; TE, 80 ms; FA, 90° and 180°; scan time, 10 min; and slice thickness 0.7 mm. T2-weighted MR images were obtained after the administration of NPs at 0, 10, and 30 min for subcutaneous injection and 1, 6, and 24 h for intravenous injection.

### 2.12. In Vivo Hyperthermia

Tumor-bearing mice were prepared as described for the in vivo MRI experiments. At day 5 post-implantation, mice were subcutaneously injected with Fe_3_O_4_/Cur@ALG NPs at a concentration of 300 µg per tumor. The injected mice were randomly divided into two groups: those subjected to AMF irradiation (Fe_3_O_4_/Cur@ALG + AMF) and those without AMF (Fe_3_O_4_/Cur@ALG). Control tumor-bearing mice were established without NP injection. A field with a 70 Oe amplitude and 178 kHz frequency was applied to the AMF mice for 40 min, starting at 15 min after injecting MNPs into the mice. Each therapy consisted of three sessions of AMF irradiation, with a 48 h break between irradiations. The treatment efficiency was described by observing the tumor volume *V*, which was calculated as follows [35]:

$$V=0.5\times a\times b^{2}\;\left({\mathrm{mm}}^{3}\right)$$

where *a* is the tumor length and b is the tumor width. Tumor measurements were performed using a caliper every two days for a total of 26 days from the day of NP injection. The tumors were photographed using a ruler at the same magnification.

### 2.13. Statistical Analysis

All statistical analyses were performed using GraphPad Prism software (version 9.1.0). Student’s *t*-test was used to test the significance of the experimental data. Statistical significance was set at *p* < 0.05.

## 3. Results

### 3.1. Structural and Morphological Analysis of the Fabricated Nanoparticles

**Figure 1:**
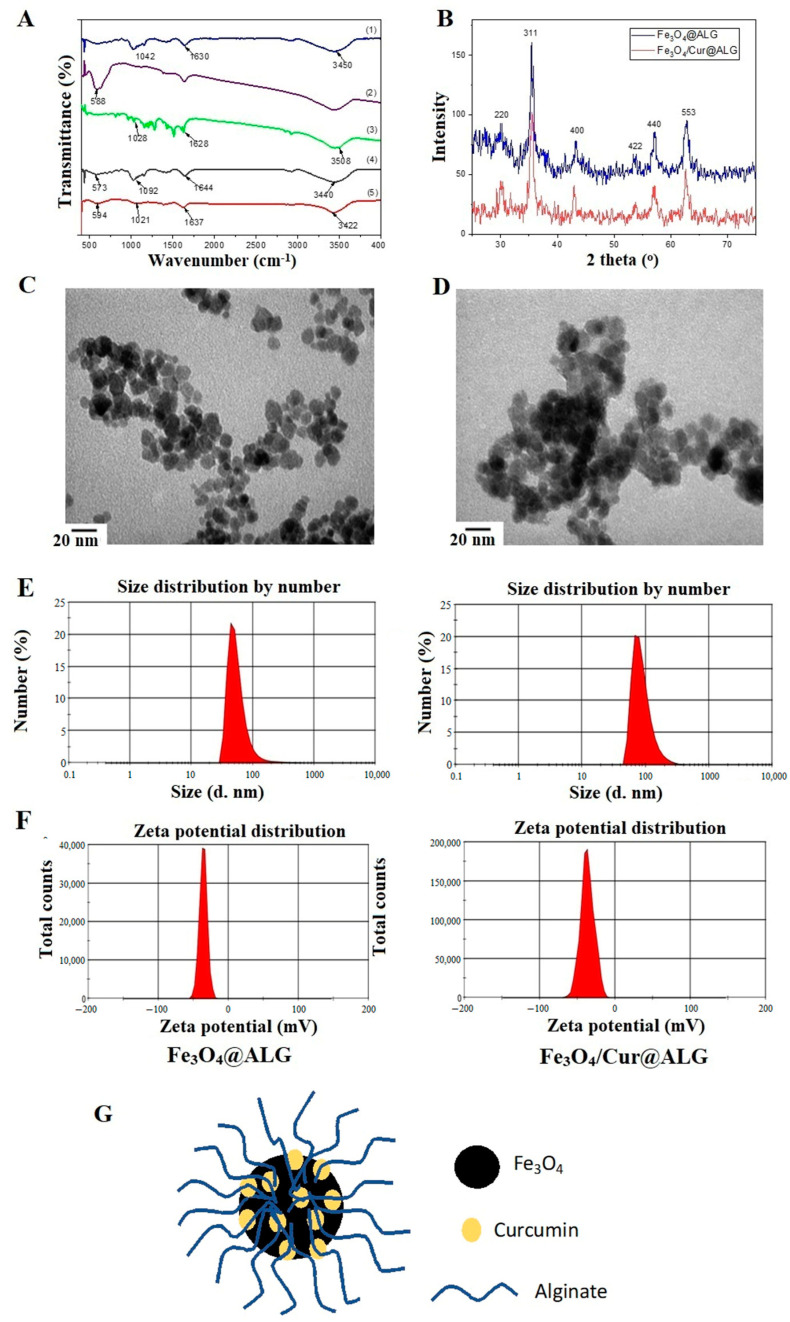
Structural and morphological analysis of NPs. (**A**) FTIR spectra of (1) alginate, (2) Fe_3_O_4_ nanoparticles, (3) curcumin, (4) Fe_3_O_4_@ALG, and (5) Fe_3_O_4_/Cur@ALG. (**B**) X-ray diffraction pattern of the Fe_3_O_4_@ALG and Fe_3_O_4_/Cur@ALG systems. (**C**,**D**) TEM images and (**E**,**F**) size and zeta potential distribution of the Fe_3_O_4_@ALG and Fe_3_O_4_/Cur@ALG nanosystems, respectively. (**G**) The schematic structure of Fe_3_O_4_/Cur@ALG nanoparticles.

In the FTIR spectra of the Fe_3_O_4_@ALG and Fe_3_O_4_/Cur@ALG systems, all typical oscillations of the functional groups in Fe_3_O_4_ nanoparticles, curcumin, and polymer alginate appear (Figure 1A). In detail, the carbonyl band (-C=O ester stretch) at 1630 cm^−1^, the -OH stretching vibration at 3450 cm^−1^, and the antisymmetric C-O-C stretch of polymer alginate at 1042 cm^−1^ shift to 1644, 3440, and 1092 cm^−1^ in the FTIR spectrum of the Fe_3_O_4_/Cur@ALG system, respectively. Moreover, the Fe-O vibration of the iron oxide nanoparticles shifts to 573 cm^−1^, confirming a good bond between the polymer alginate and the Fe_3_O_4_ core layer. In addition, these characteristic peaks shift in the spectrum of Fe_3_O_4_/Cur@ALG, and the corresponding wavenumbers are 1637 cm^−1^ (C=O), 3442 cm^−1^ (-OH), 1021 cm^−1^ (C-O-C), and 594 cm^−1^ (Fe-O). Moreover, peaks characterizing the C=O bond, O-H band, and C-O-C stretch of curcumin are also observed in the Fe_3_O_4_/Cur@ALG spectrum. These results reveal that curcumin is successfully incorporated into the Fe_3_O_4_@ALG nanosystem.

From the XRD diagram, the peaks are obvious, the main spectral clusters have high intensities, and the background lines of the samples are relatively flat and do not mix with strange phases, indicating that the nanosystems produced are of good quality. The peaks at angular positions 2θ = 31°, 36°, 43°, 53°, 57°, and 63° correspond to the (220), (311), (400), (422), (440), and (533) planes of the iron oxide nanoparticle nanocrystals, respectively (Figure 1B). These results also suggest that, after coating with alginate and loading with curcumin, the crystal structure of the iron oxide nanoparticles does not change, as shown by the matching of characteristic peaks at the characteristic crystal surfaces of Fe_3_O_4_ nanocrystals. From the XRD scheme, we can calculate that the average size of the iron oxide nanoparticles is approximately 15 nm according to Scherrer’s formula D = Kλ/(β cos θ), where K is a constant (K = 0.9 for Cu-Kα), λ is the wavelength (0.15406 nm for Cu-Kα), and β is the full width at half maximum of the (311) peak [36]. 

The morphology of the materials was observed using TEM. Figure 1C,D show TEM images of the Fe_3_O_4_@ALG and Fe_3_O_4_/Cur@ALG systems. The nanoparticles are round and monodispersed with a diameter of approximately 12–15 nm, and their distribution is relatively uniform.

Furthermore, we evaluated the particle size in a liquid medium and the zeta potential of Fe_3_O_4_@ALG and Fe_3_O_4_Cur@ALG. The liquid samples were analyzed using dynamic laser scattering spectroscopy on a Zetasizer potentiometer. Distribution diagrams of the particle size and zeta potential are shown in Figure 1E,F, respectively. The schematic structure of Fe_3_O_4_/Cur@ALG nanoparticles is shown in Figure 1G. The hydrodynamic dimensions of Fe_3_O_4_@ALG and Fe_3_O_4_/Cur@ALG are 92 and 98 nm, respectively. These sizes are significantly larger than the particle sizes measured from the TEM images; however, this could be explained by the polydispersity of the polymer alginate. According to classical Rayleigh theory, the intensity of the scattered light depends on the square of the volume of the scattering substance, resulting in larger particle sizes in the multiple dispersion state [37]. In addition, the zeta potentials of both the Fe_3_O_4_@ALG and Fe_3_O_4_/Cur@ALG nanosystems are −41.3 mV and −38.5 mV, respectively. These zeta potential values are due to the binding between the carboxyl groups of alginate and the Fe_3_O_4_ core, as well as the large zeta potential that creates an electrostatic repulsion between nanoparticles with the same charge. These zeta potentials prove that they have good stability for possible biomedical applications. 

### 3.2. Acute and Sub-Chronic Toxicity of Fe_3_O_4_/Cur@ALG NPs

**Figure 2:**
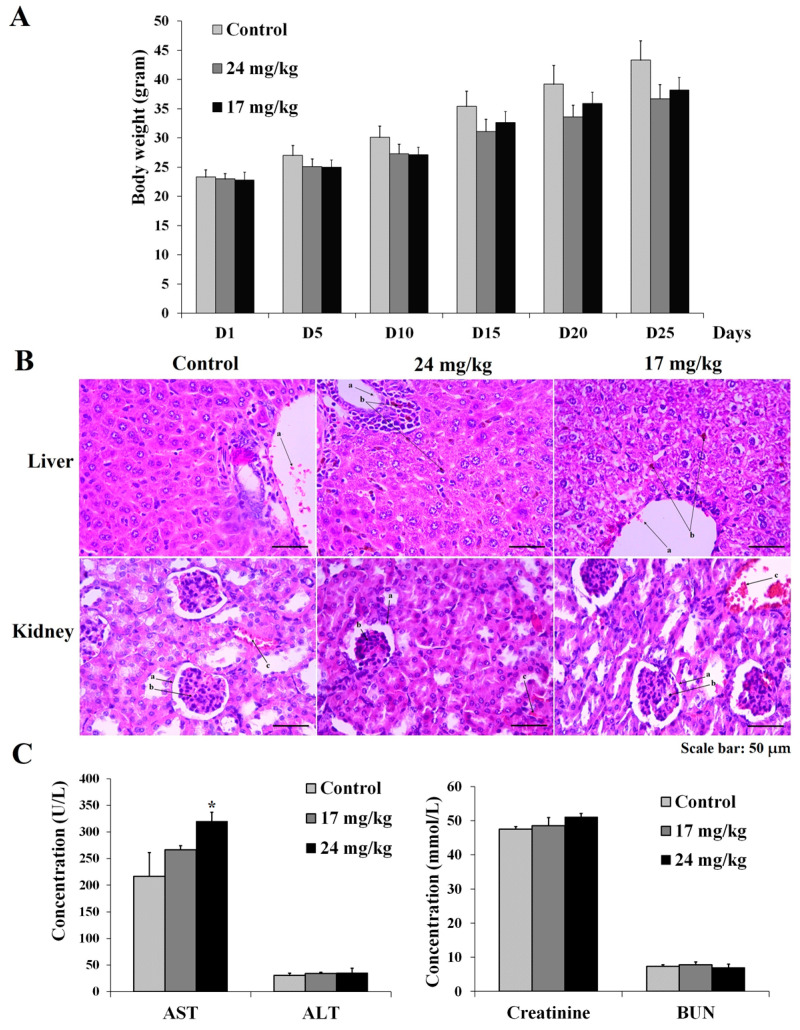
The acute and sub-chronic toxicity of Fe_3_O_4_/Cur@ALG NPs on Swiss mice. (**A**) The body weight of mice during 30 days of treatment via intravenous injection with 24 mg/kg or 17 mg/kg NPs every day. (**B**) Histological analysis of liver and kidney tissues from the control and treated mice. In the liver: (a) red blood cells; (b) magnetic NPs. In the kidney: (a) Bowman’s capsule; (b) glomerulus; (c) red blood cells. (**C**) The concentration of functional indicators in the liver and kidneys. Data are presented as the mean ± SD (n = 10). * *p* ˂ 0.05.

The acute toxicity assay of Fe_3_O_4_/Cur@ALG NPs was performed at five doses ranging from 80 to 120 mg/kg. After 72 h after the intravenous injection, none of the mice in the five treatment groups died. Moreover, there were no differences between the control and treated groups in terms of performance and behavior (Figure 2A). This result indicates that Fe_3_O_4_/Cur@ALG NPs does not elicit acute toxicity in mice in the range of the tested doses. Hence, we chose two doses, 24 mg/kg and 17 mg/kg, equal to 1/5 and 1/7 of 120 mg/kg, respectively, which was the highest dose used in the acute toxicity test and the sub-chronic toxicity test. In the two treatment groups, each mouse was continuously injected with 24 or 17 mg/kg/day for 30 days. The body weight of the mice was measured every five days, and the functional activities of the liver and kidney were evaluated at the end of the experiment. The results show that there is a modest decline in the body weight of the treated mice (2–10%) compared to that of the control; however, the difference is not significant (*p* > 0.05) (Figure 2A). Notably, two mice died, one on day 15 and one on day 20, in both treatment groups (24 or 17 mg/kg/day, respectively), accounting for 20% of the deaths. Histological analysis indicates that Fe_3_O_4_/Cur@ALG NPs are present in small amounts in the liver but not in the kidneys. For histological analysis, we observed the nanoparticle conglomerate-like structures present in the liver, but not kidney tissues in the mice treated with NPs. In addition, the histological structures of livers changes compared to those in the control group, with the appearance of vacuolated hepatocytes (Figure 2B). In addition, biochemical assay data indicate that both AST and ALT values are higher in the treated mice than in the control mice, with a significant difference in AST values (*p* < 0.05), whereas the levels of BUN and creatinine do not change significantly (Figure 2C). Thus, the Fe_3_O_4_/Cur@ALG NPs likely accumulate in the liver, causing an increase in transaminase levels.

### 3.3. The Lifetime of Fe_3_O_4_/Cur@ALG NPs in the Blood

**Figure 3:**
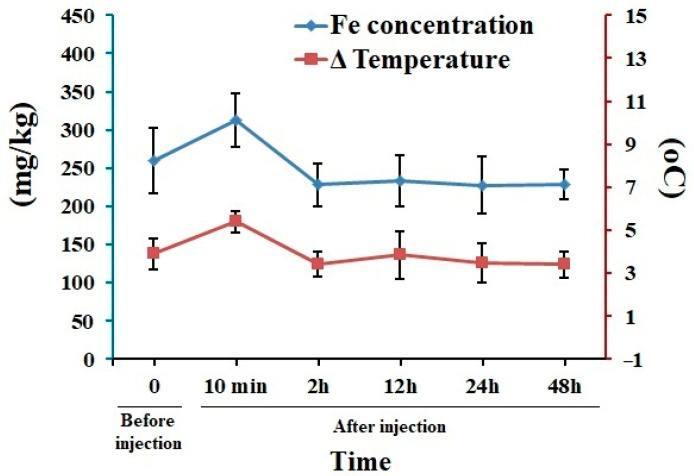
The lifetime of Fe_3_O_4_/Cur@ALG NPs in the blood. Data from the magnetic and AAS analyses reveal changes in the temperature (blue line) and Fe concentration (red line) over time after the intravenous injection of magnetic NPs. Data are presented as the mean ± SD (n = 5).

We determined the presence of Fe_3_O_4_/Cur@ALG NPs in the blood of mice using magnetic inductive heating (MIH) measurements and AAS. After intravenous injection of 1000 µg magnetic NPs, the mice were placed in an AMF of 178 kHz and 80 Oe. By measuring the change in temperature in the blood samples drawn from mice blood vessels over time, we find a rise in temperature in the blood sample in the treated mice at 10 min immediately after the injection. The temperature declines to a comparable value before NP application, at 2 h after injection, and at every time point after that. Of note, the indirect measurement of Fe concentrations by temperature change could be affected by many factors in the blood. A standard curve with the known concentrations of Fe should be used to avoid this problem. However, similar results are obtained via the AAS method; the concentration of Fe in the blood samples of the treated mice increases 10 min after injection and then decreases to the value of the control (Figure 3). Taken together, these results indicate that Fe_3_O_4_/Cur@ALG NPs are cleared from the blood within a short time of approximately 10 min. 

### 3.4. MRI Enhancement

**Figure 4:**
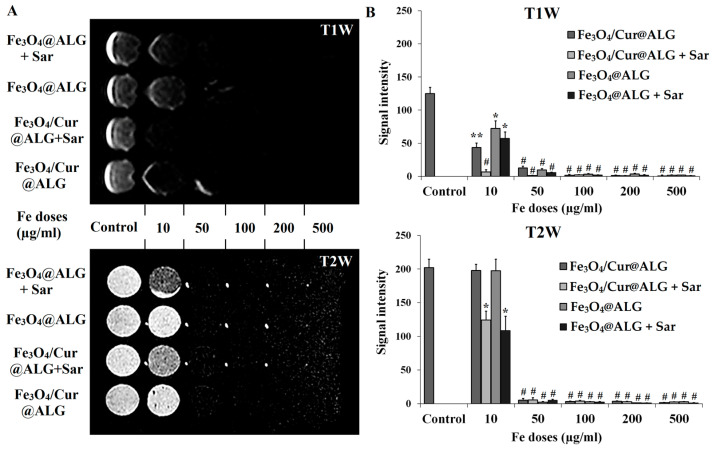
MRI contrast enhancement of Fe_3_O_4_/Cur@ALG and Fe_3_O_4_@ALG NPs in vitro. (**A**) T1- and T2-weighted MR images (T1W and T2W, respectively) were obtained in the presence of the two types of NPs. Fe_3_O_4_/Cur@ALG and Fe_3_O_4_@ALG NPs at different concentrations of Fe from 10–500 µg/mL mixed with 1% agarose only and 1% agarose solution containing sarcoma 180 cells. (**B**) Quantitative analysis of MR signal intensity under different conditions. Data are presented as the mean ± SD of triplicate experiments. * *p* ˂ 0.05; ** *p* < 0.01; ^#^ *p* < 0.001. Sar: sarcoma cells.

For the MRI contrast enhancement assay, we used two conditions: Fe_3_O_4_/Cur@ALG NPs mixed with (1) 1% agarose and (2) 1% agarose solution with cells. Condition 2 mimicked the environment surrounding the nanoparticles after they were injected into the body. Figure 4A shows clear differentiation in the dark signal when comparing the treated wells to the control, even from the lowest tested Fe concentration (10 µg/mL). The dark-scale color progressively darkens with increasing concentrations of Fe_3_O_4_. In addition, the dark signal in the sarcoma-180-cell-containing agarose solution is stronger than in the agarose solution alone. Quantitative analysis of the MR images reveals that the MR signal intensity is significantly higher in control than in the nanoparticle-treated wells and that a higher Fe concentration reduces the signal intensity of the MR images (*p* < 0.05) (Figure 4B). In the current study, we also observe that the MRI contrast is better enhanced in the T2 than in the T1 images (*p* < 0.05).

**Figure 5:**
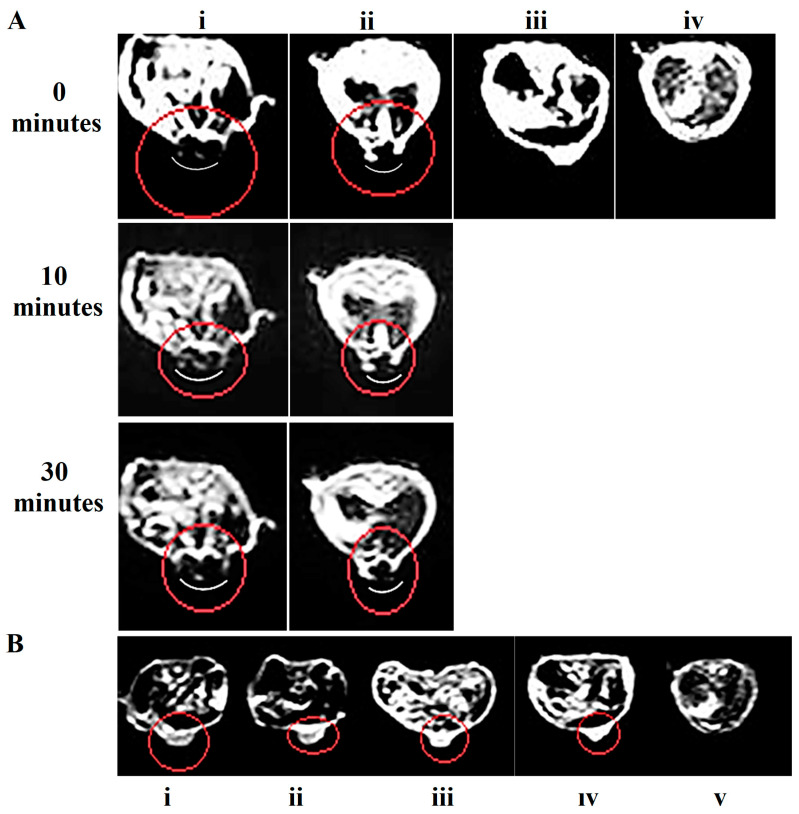
MRI contrast enhancement of Fe_3_O_4_/Cur@ALG NPs in vivo. T2-weighted MR images were obtained with the parameters: TR/TE 3000/80 ms, 180-degree flip angle, and “head-to-tail” axis scanning. (**A**) Fe_3_O_4_@ALG NPs were directly injected into the tumor at two concentrations, 250 µg/0.5 cm^3^ (**i**) and 50 µg/0.5 cm^3^ (**ii**); the controls include a tumor control without nanoparticles (**iii**) and without tumors (**iv**). (**B**) Fe_3_O_4_/Cur@ALG NPs were injected via the tail vein of the mice at a concentration of 1000 µg/mouse. MR images were taken at 1 h (**i**), 6 h (**ii**), and 24 h (**iii**) post-injection. MR images of the tumor control without nanoparticles (**iv**) and the control without tumors (**v**). Red circles: the tumor region.

Based on the results of in vitro MRI, we chose a T2-weighted scan for in vivo imaging. We performed MRI on mice bearing sarcoma tumors on day 5 after cancer cell implantation when the average tumor size was 100 mm^3^. Fe_3_O_4_/Cur@ALG NPs were directly applied to the tumor at different concentrations, 50 µg/0.5 cm^3^ and 250 µg/0.5 cm^3^, and then MR scanning was performed at 0, 10, and 30 min after the injection. The tumor regions gradually darken, and the dark areas progressively enlarge throughout the entire tumor after injection. However, it is difficult to identify the tumor site in the untreated mice. In addition, the higher the concentration of Fe, the darker the signal. Interestingly, even with a low concentration of Fe (50 µg/0.5 cm^3^), the contrast of the MR images is still enhanced (Figure 5A).

However, when the NPs are intravenously injected, we observe no difference in the image contrast between the control and treated mice (Figure 5B). These results are inconsistent with the biodistribution results (Figure 2), in which the nanoparticles are already cleared or are not yet focused on the tumor. Nevertheless, our results suggest that the Fe_3_O_4_/Cur@ALG NPs can be used for imaging-guided therapy. For MRI diagnosis, MNPs must be modified to enhance the permeability and retention effect so that they can be used intravenously. To determine the time when MNPs remain in the tumor, images were taken 1–2 h post-MNP injection. MRI prior to MNP injection should also be performed for every tested tumor to precisely determine the effect of MNPs on image contrast enhancement.

### 3.5. Bioeffects of Fe_3_O_4_/Cur@ALG NPs on Cancer Cells

**Figure 6:**
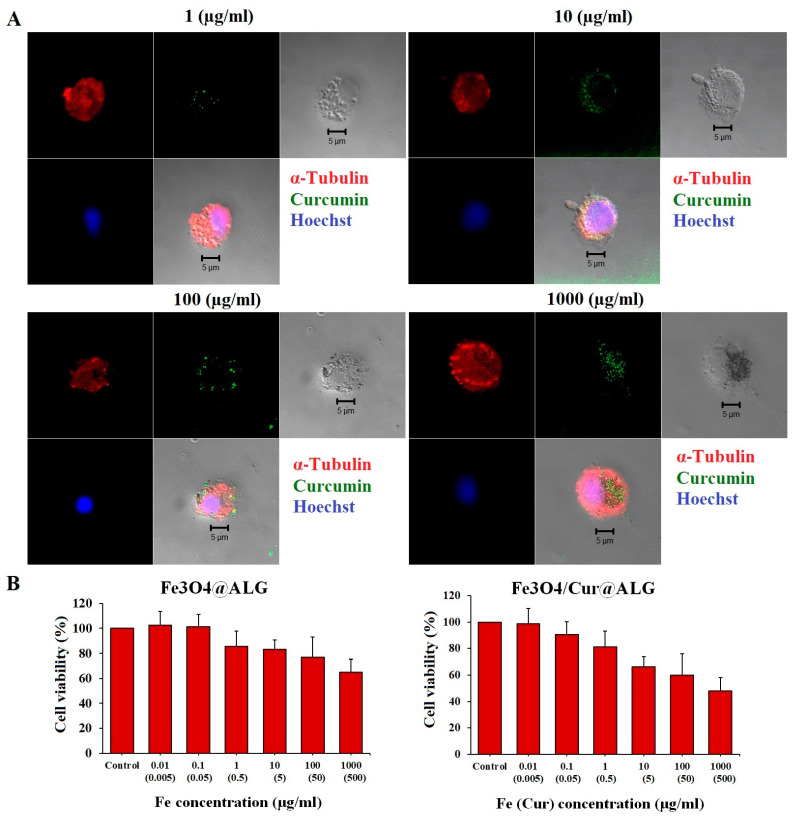
Cytotoxicity of the Fe_3_O_4_/Cur@ALG and Fe_3_O_4_@ALG NPs on sarcoma 180 cells. (**A**) The uptake of Fe_3_O_4_/Cur@ALG into sarcoma 180 cells can be seen based on the green autofluorescence signal of curcumin after 48 h of incubation with different concentrations of Fe. Cell nuclei were stained with Hoechst, and the cytoskeleton was visualized using microtubule staining (in red). (**B**) The proliferation of sarcoma 180 cells after 48 h of incubation with the two types of NPs. Data are presented as the mean ± SD of triplicate experiments. Scale bar: 5 µm.

We observed the penetration of Fe_3_O_4_/Cur@ALG NPs into sarcoma-180 cells based on the characteristic autofluorescence of curcumin after 48 h of incubation. The higher the NP concentration, the more particles that are in the cytoplasm (Figure 6A). It is worth noting that cells treated with high concentrations of Fe show many blebs, indicating cell membrane disruption (Figure 6A).

Data analysis of the cell viability assay shows that Fe_3_O_4_/Cur@ALG NPs are cytotoxic to sarcoma 180 cells only at high doses. Nearly 49% of cell proliferation is inhibited at a dose of 1000 µg/mL Fe, corresponding to 500 µg/mL curcumin (Figure 6B). The IC50 value of the nanoparticles is 897 ± 38 µg/mL for Fe, corresponding to 444.8 ± 42 µg/mL for curcumin. In addition, Fe_3_O_4_@ALG is low in toxicity to sarcoma 180 cells, with cell growth at 1000 µg/mL Fe being 65 ± 3.5% (Figure 6B). These results indicate that cytotoxicity increases in the presence of curcumin.

**Figure 7:**
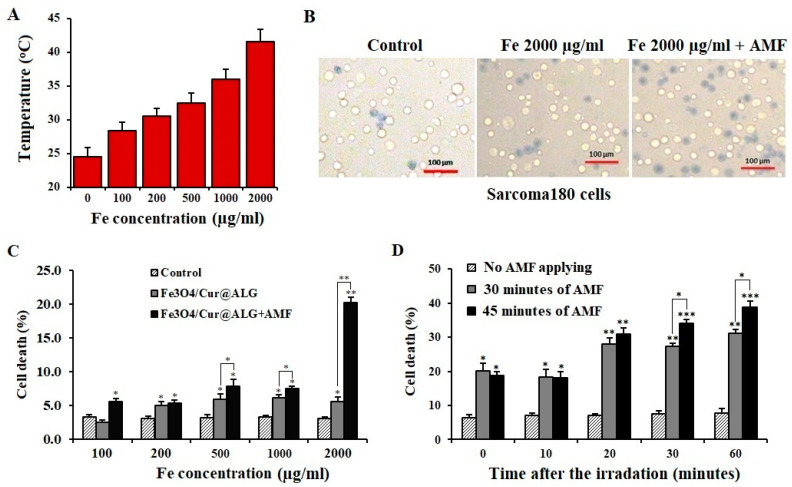
In vitro magneto-inductive heating of Fe_3_O_4_/Cur@ALG NPs induces the death of sarcoma 180 cells. (**A**) The temperature (°C) values reached after stimulating the Fe_3_O_4_/Cur@ALG NPs with an alternative magnetic field (AMF). (**B**) Images of sarcoma 180 cells stained with Trypan blue after incubation with 2000 µg/mL Fe_3_O_4_/Cur@ALG NPs and AMF. (**C**) Percentages of cell death (%) after 30 min of AMF irradiation. (**D**) Cell death (%) at different time points after finishing the 30- and 45-min AMF protocols after treatment with 2000 µg/mL Fe_3_O_4_. Data are presented as the mean ± SD of triplicate experiments. * *p* ˂ 0.05; ** *p* < 0.01; *** *p* < 0.001.

Magnetic heating treatment significantly reduces the viability of sarcoma 180 cells (*p* < 0.05) (Figure 7A) and increasing the time of field irradiation further induces cell death (Figure 7B). Interestingly, the cell death rate continues to increase significantly over time after the completion of irradiation (*p* < 0.05) (Figure 7C). We also find that cell death increases through the domino effect after the heating process is stopped. At 60 min after heating, cell death increases to 31.1% and 38.8% after AMF treatment for 30 min and 45 min, respectively (Figure 7D). This means that the death of sarcoma 180 cells still occurs even when the alternating field is stopped. Hyperthermia can induce apoptosis in cancer cells through heating [38], and curcumin is proven to initiate programmed cell death [23,29]. Under these conditions, many apoptotic cells do not die continuously during the heating treatments but after the process finishes. Therefore, to assess the effectiveness of hyperthermia accurately, the number of dead cells must be determined over time. Taken together, these results suggest that Fe_3_O_4_/Cur@ALG NPs have the potential to be used as inductive heating agents for hyperthermia-induced cancer cell death. These promising results from the in vitro assays encouraged us to evaluate the bioactivity of Fe_3_O_4_/Cur@ALG NPs in vivo.

**Figure 8:**
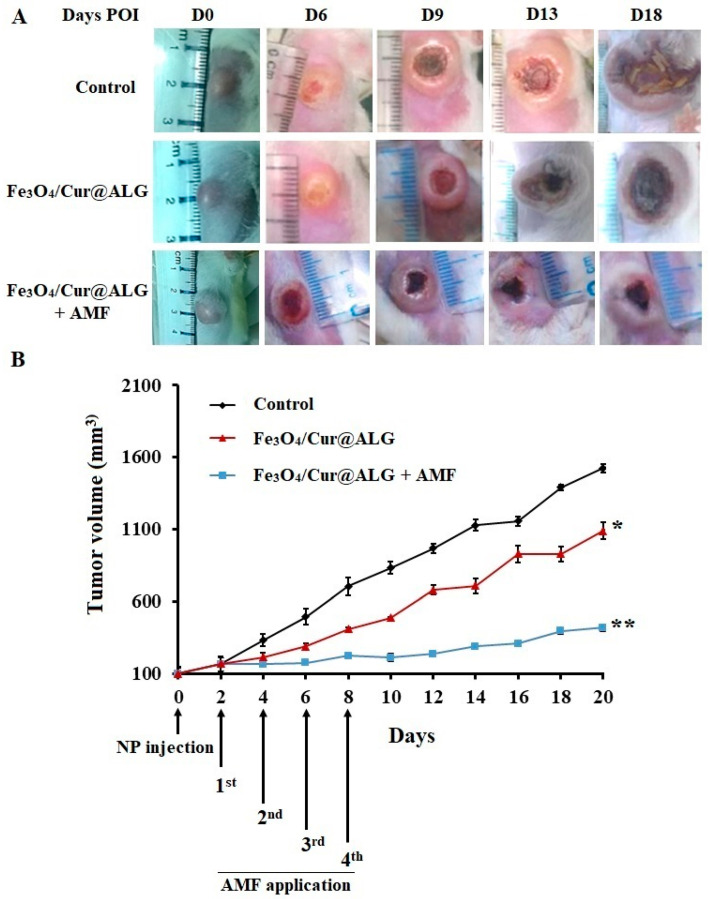
Magnetic hyperthermia treatment in sarcoma 180 tumor-bearing mice. (**A**). Images of tumors by days post-injection (POI) in the three groups of mice: the control without treatment; the mice injected with Fe_3_O_4_/Cur@ALG NPs; and the mice injected with Fe_3_O_4_/Cur@ALG NPs subjected to an alternative magnetic field (AMF) (Fe_3_O_4_/Cur@ALG + AMF). (**B**) The tumor growth curve during the in vivo experiment. After four treatments at 48 h intervals, the mice were continuously observed, and the volume of tumors was measured every two days. Data are presented as the mean ± SD (n = 10). * *p* ˂ 0.05; ** *p* < 0.01.

Hyperthermia treatment was performed on Swiss mice bearing sarcoma 180 tumors. Next, Fe_3_O_4_/Cur@ALG NPs were directly injected at a concentration of 250 µg/0.5 cm^3^ into the tumor on day 5 post-transplantation and subjected to an AMF of 178 kHz and 80 Oe. Treatment was repeated four times at 48 h intervals. At this point, the average tumor size was 100 ± 22.3 mm^3^. We observe the smallest significant differences in the size of tumors in mice treated with NPs and AMF, followed by those treated with NPs only, and then the control (Figure 8A). There is a strong inhibition of tumor growth in mice treated with Fe_3_O_4_/Cur@ALG and AMF, especially from day 0 to day 8 post-injection (POI) (Figure 8B). At the end of the experiment (day 20 POI), the size of the tumors in the NPs- and AMF-treated mice increases by approximately 400% compared to day 0 POI, while tumor size increases by 1600% compared to the control (Figure 8B). Interestingly, in NP-treated mice, the tumor volume is smaller than that in the untreated control, with the average size increasing by 1100% on day 26 POI (Figure 8B).

## 4. Discussion

For applications in humans, it is necessary to comprehensively evaluate the toxicity of the nanoparticles. Toxicity tests may help determine whether the nanoparticle concentrations are sufficiently high to cause adverse effects in the treated organisms. The promising results of Fe_3_O_4_/Cur@ALG NPs in MRI and hyperthermia treatment prompted us to perform acute and sub-chronic toxicity tests. In the acute lethality test, no mice died after 72 h of treatment, even with the highest dose of 120 mg/kg. This indicates that the nanoparticles do not induce acute lethality in mice at this dose. We also chose two doses corresponding to 1/7 and 1/5 of the highest dose to test for chronic toxicity. The purpose of the sub-chronic toxicity test was to measure the effects of exposure to relatively low, repeated, and less toxic concentrations of compounds over a prolonged period. After intravenous injection every day for 30 days, corresponding to total doses of 720 and 530 mg/kg/mouse, two out of ten mice died. However, there are no statistically significant differences in body weight or daily activity between the control and treated groups (*p* < 0.05). Histological analysis shows that the liver tissue structures change in the injected mice compared to the control, with the presence of vacuolated hepatocytes. In addition, few nanoparticle conglomerate-like structures are observed in the liver tissue. Interestingly, these structures are detected only in the NPs-treated samples but not the control, suggesting that they are the accumulation of NPs. Specific staining, such as Prussian blue, would be performed to confirm the presence of these magnetic nanoparticles. Since we analyzed the histological structure of the liver right after the last injection, it may be too short a time for the body to clear the nanoparticles. A longer time of assessment after the NP injection should be performed to determine the accumulation of the nanoparticles in the liver. To evaluate liver function, we measured ALT and AST levels, which are well-known indicators for functional tests of the liver [33]. The obtained results agree with the histological analysis, wherein there is an increase in the levels of these enzymes in the treated mice compared to the control, significant to AST (*p* < 0.05). However, the ALT levels in the treated mice are still in the normal range (25–60 U/L) [33]. If AST levels are three times the upper limit of the normal range (39–262 U/L), it indicates abnormal liver function [33]. The changes observed in our study are significantly lower than this threshold. Still, the increase in the level of both ALT and AST together with the liver structural change may indicate damage to the liver. This is consistent with the previous literature about the liver toxicity of magnetic nanoparticles [39]. Hence, the long-term safety of this nanosystem should be further investigated. 

Previous studies reported the toxicity of magnetic iron oxide NPs (IONPs) in vivo [33]. Our results are similar to those of Jain et al., who report that magnetic NPs increase the levels of liver enzymes, but they remain in the normal range. In a study by Feng et al., polyethyleneimine-coated IONPs exhibited dose-dependent lethal toxicity, with 100% animal mortality at the tested concentration of 5 mg/kg. In contrast, there were no dead mice in the PEGylated-coated IONPs group at the same Fe concentration [33]. It should be noted that in these studies, the NPs were injected only once per treatment, which was much less frequent than that in our study (30 times in 30 days). Regarding iron concentrations, the levels of iron in the blood of the treated mice returns to normal 10 min after injection, indicating the quick clearance of NPs in the blood. The lifetime of our nanoparticles is shorter than most of reported NPs [40], and this could be due to the phagocytosis process [41]. Taken together, our data clearly indicate that our Fe_3_O_4_/Cur@ALG NPs are not acutely toxic, and do not cause adverse effects on the kidney, but alter the liver structure when tested at high doses for over 30 days.

Regarding MRI enhancement, the time at which the nuclei return from an excited state to an equilibrium state is called the relaxation time (T), which depends on the proton density in the tissues [42]. Magnetic NPs are considered superparamagnetic, which have the potential to reduce the T2 relaxation time and increase negative image contrast [20,43]. In the current study, we also observe that the MRI contrast is better enhanced in the T2 than in the T1 images. The MRI data for Fe_3_O_4_/Cur@ALG show results compatible with their characteristics as superparamagnetic iron oxide NPs. In the presence of curcumin, the nanosystem still has the ability to enhance the image contrast, with the MR signal intensity significantly reduced even at the smallest Fe concentration used in the experiment (10 µg/mL). In addition, when NPs are applied for MRI diagnosis, their interaction with the cells of the body must be considered, because they can change the MRI signal. The results of our study indicate that the presence of cells does not affect the T2 MRI signal of Fe_3_O_4_/Cur@ALG. Our results are compatible with those of other superparamagnetic iron oxide NPs, which have also been reported to increase MRI contrast [21,42]. Moreover, our data are consistent with the most recent report by Thong et al., who reported that the T2 contrast mechanism is dominant for this type of magnetic nanoparticle [44]. In addition, Fe_3_O_4_/Cur@ALG NPs exhibit an MRI effect when directly injected into mice. At Fe concentrations of 50 and 250 µg/0.5 cm^3^, the NPs increase the negative contrast of MR images at the tumor site, with the darkening signal increasing with the time and density of NPs. It is worth noting that the dark region in MRI could be caused by the necrotic core in the tumor. Thus, we chose five-day-old tumors in the early logarithmic growth phase. Moreover, the MRI images of the untreated mice reveal no necrotic cores in their tumors. Thirty minutes after nanoparticle injection, the dark signal is maintained, indicating that the nanoparticles are still present at the tumor site. Our results are consistent with those of Sun et al., who report that the iron oxide nanoparticle-immobile alginate nanogels remain in tumors no shorter than 2 h after injection [45]. 

A previous study by Devkota et al. showed that Fe_3_O_4_/Cur@ALG NPs could be used as an inductive heating agent when applied in an alternating magnetic field [12]. In this study, we aimed to determine whether this ability is present in the mixture with cells and, more importantly, whether NP-induced hyperthermia can kill cancer cells. In addition, we chose Fe_3_O_4_/Cur@ALG NPs, but not Fe_3_O_4_@ALG NPs, because the former showed greater cytotoxicity in sarcoma cells in vitro than the latter. The temperature rise of the MNPs was examined with a concentration range of MNPs from 100 to 2000 µg/mL. The magnetic field strength was fixed at 80 Oe and the frequency was set at 178 kHz. At the highest MNP concentration, the temperature is 41.5 °C after 30 min of AMF exposure (Figure 7A). This temperature is lower than that reported for the same Fe concentration in the above-mention study [12]. This difference may be due to the presence of cancer cells in the heated solutions. Previous studies also indicate that the temperatures of cell-containing solutions are always lower than that of solutions consisting of magnetic NPs only [1,46,47]. Even so, a temperature of 41.5 °C can still potentially kill cancer cells, with 20% of sarcoma cells dying (Figure 7B,C). In some reported studies, although the temperature was higher than 45 °C, the cancer cell viability after 30 min of AMF application was less than that in our experiment. The higher rate of cell death in the treatment compared to other studies can be explained by the anti-cancer activity of curcumin. Previous studies showed that MIH could release curcumin from nanoconjugates [9,47] so that it could kill cancer cells.

The results of hyperthermia treatment in tumor-bearing mice are consistent with those of the in vitro tests. The volume of sarcoma 180 tumors is reduced in the heated mice, especially during the heating process (D0–D8). This effect is maintained after finishing the treatment until D14. This phenomenon clearly indicates the domino effect of hyperthermia. Although the tumors re-grow after D14, the progression rate is four times lower than that in the untreated mice. The regrowth of the tumor suggests that it is necessary to repeat the treatment after some time to obtain a more efficient anti-tumor effect. Interestingly, the tumor volume also becomes smaller in mice injected with Fe_3_O_4_/Cur@ALG NPs without heating compared to the control. This effect could be attributed to curcumin, which has been proven to have a role as an anticancer substance [7,23]. Collectively, our results reveal that Fe_3_O_4_/Cur@ALG NPs efficiently kill cancer cells in vitro and in vivo. Moreover, cancer cell death may be due to the synergetic effect of combining thermal therapy and chemotherapy with curcumin.

## 5. Conclusions

In conclusion, the Fe_3_O_4_/Cur@ALG NPs could be used in biomedical applications with very low acute toxicity on mice. However, the long-term effect on liver structure and function should be determined. The application of Fe_3_O_4_/Cur@ALG NPs for MRI and cancer treatment in both in vitro and in vivo models has also been demonstrated. These NPs can enhance MRI contrast, kill cancer cells, and inhibit tumor growth through a synergetic combination of thermal therapy and chemotherapy. We recommend that further studies on the elimination and biodistribution of these NPs be performed to successfully develop them for clinical testing.

## Data Availability

All data that support the findings of this study are included in the manuscript. Data will be made available on request. This manuscript has partly reported as a preprint on Research Square with the following link: https://www.researchsquare.com/article/rs-1843318/v1 and DOI: 10.21203/rs.3.rs-1843318/v1.
